# Arabidopsis AtPLC2 Is a Primary Phosphoinositide-Specific Phospholipase C in Phosphoinositide Metabolism and the Endoplasmic Reticulum Stress Response

**DOI:** 10.1371/journal.pgen.1005511

**Published:** 2015-09-24

**Authors:** Kazue Kanehara, Chao-Yuan Yu, Yueh Cho, Wei-Fun Cheong, Federico Torta, Guanghou Shui, Markus R Wenk, Yuki Nakamura

**Affiliations:** 1 Institute of Plant and Microbial Biology, Academia Sinica, Taipei, Taiwan; 2 Molecular and Biological Agricultural Sciences Program, Taiwan International Graduate Program, Academia Sinica, Taipei, Taiwan; 3 Graduate Institute of Biotechnology and Department of Life Sciences, National Chung-Hsing University, Taichung, Taiwan; 4 Muroran Institute of Technology, Muroran, Japan; 5 Department of Biochemistry, Yong Loo Lin School of Medicine, National University of Singapore, Singapore; 6 Life Sciences Institute, National University of Singapore, Singapore; 7 State Key Laboratory of Molecular Developmental Biology, Institute of Genetics and Developmental Biology, Chinese Academy of Sciences, Beijing, China; 8 Department of Biological Sciences, National University of Singapore, Singapore; The University of North Carolina at Chapel Hill, UNITED STATES

## Abstract

Phosphoinositides represent important lipid signals in the plant development and stress response. However, multiple isoforms of the phosphoinositide biosynthetic genes hamper our understanding of the pivotal enzymes in each step of the pathway as well as their roles in plant growth and development. Here, we report that phosphoinositide-specific phospholipase C2 (AtPLC2) is the primary phospholipase in phosphoinositide metabolism and is involved in seedling growth and the endoplasmic reticulum (ER) stress responses in *Arabidopsis thaliana*. Lipidomic profiling of multiple *plc* mutants showed that the *plc2-1* mutant increased levels of its substrates phosphatidylinositol 4-phosphate and phosphatidylinositol 4,5-bisphosphate, suggesting that the major phosphoinositide metabolic pathway is impaired. AtPLC2 displayed a distinct tissue expression pattern and localized at the plasma membrane in different cell types, where phosphoinositide signaling occurs. The seedlings of *plc2-1* mutant showed growth defect that was complemented by heterologous expression of *AtPLC2*, suggesting that phosphoinositide-specific phospholipase C activity borne by AtPLC2 is required for seedling growth. Moreover, the *plc2-1* mutant showed hypersensitive response to ER stress as evidenced by changes in relevant phenotypes and gene expression profiles. Our results revealed the primary enzyme in phosphoinositide metabolism, its involvement in seedling growth and an emerging link between phosphoinositide and the ER stress response.

## Author Summary

Plant growth requires continuous signal transduction in response to ever-changing environment. Phosphoinositides represent primary lipid-derived signals that are involved in various plant responses to surrounding environment. However, enzymes that determine the complex phosphoinositide molecular profiles remained elusive due to the existence of a number of candidate genes for each step. Here, in *Arabidopsis thaliana*, we found AtPLC2 as the enzyme that is decisive in phosphoinositide metabolism by analytical lipidomics of the gene knockout study. Functional characterization of AtPLC2 knockout plant enabled us to find two novel roles of phosphoinositides: requirement in seedling growth and ER stress tolerance. Because economically important environmental stresses such as salinity and temperature upshift cause ER stress, our findings may open up a new avenue in addressing ER stress responses via phosphoinositide signaling.

## Introduction

**Fig 1:**
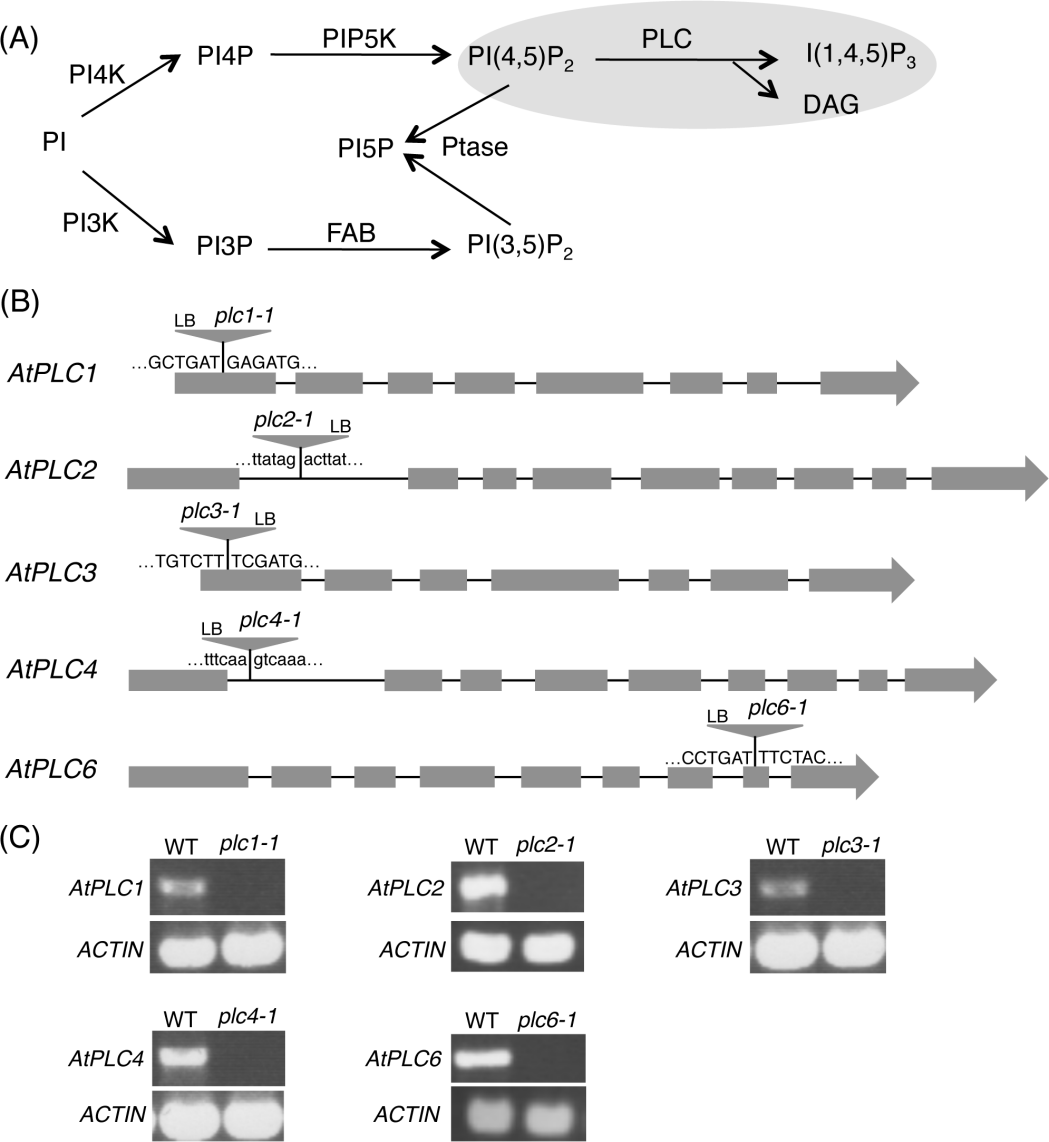
Phosphoinositide-specific phospholipase C (PI-PLC) in phosphoinositide metabolism. (A) Proposed phosphoinositide metabolic pathways in Arabidopsis. PI, phosphatidylinositol; PI3K, PI 3-kinase; PI4K, PI 4-kinase; PIP5K, PI4P 5-kinase; Ptase, phosphoinositide phosphatase; DAG, *sn*-1,2-diacylglycerol; FAB, formation of aploid and binucleate cells; I(1,4,5)P_3_, inositol 1,4,5-trisphosphate. (B) Schematic representations of *AtPLC1* (At5g58670), *AtPLC2* (At3g08510), *AtPLC3* (At4g38530), *AtPLC4* (At5g58700) and *AtPLC6* (At2g40116). Gray boxes and lines represent exons and introns, respectively. The positions of T-DNA insertions of *plc1-1*, *plc2-1*, *plc3-1*, *plc4-1*, and *plc6-1* are indicated by triangles. (C) RT-PCR analysis of gene transcripts for *AtPLC1* (1.7 kb), *AtPLC2* (1.8 kb), *AtPLC3* (1.7 kb), *AtPLC4* (1.8 kb), and *AtPLC6* (1.9 kb) in the wild-type plants and the mutants. The Ws plants were used as the wild type for *AtPLC2*. *Actin* (0.5 kb) was used as a control.

The phosphoinositides are phosphorylated derivatives of phosphatidylinositol, which represent a minor portion of phospholipids but a major role in lipid signaling from bacteria to seed plants or mammals [1]. The final step of phosphoinositide metabolism is the hydrolysis of phosphatidylinositol 4,5-bisphosphate [PI(4,5)P_2_] into inositol 1,4,5-trisphosphate (IP_3_) and *sn*-1,2-diacylglycerol (DAG) that is catalyzed by phospholipase C (PLC) (Fig 1A). This reaction is a crucial step in achieving signal transduction: IP_3_ triggers Ca^2+^ influx and DAG kinase readily converts DAG to phosphatidic acid (PA), which serves as a lipid second messenger to fulfill distinct roles of signal transduction in plants [2]. Therefore, investigating function of PLC has utmost importance in understanding the entire phosphoinositide signaling.

Two types of PLC are known in Arabidopsis: non-specific PLC (NPC), which hydrolyzes abundant phospholipids such as phosphatidylcholine (PC), phosphatidylethanolamine; and phosphoinositide-specific PLC (PI-PLC), which is specific to PI(4,5)P_2_ and its related derivatives [3]. The NPC family has six isoforms, NPC1 to NPC6. For example, NPC4 and NPC5 are involved in membrane lipid remodeling in response to phosphate starvation [3,4]. On the other hand, nine genes are annotated as Arabidopsis PI-PLC, designated *AtPLC1* to *AtPLC9* [5,6]. *AtPLCs* are involved in broad range of stress-induced lipid signaling [1,7]. A majority of the *AtPLC* genes are transcriptionally induced by various environmental stimuli such as salt, cold and dehydration [7–11]. *AtPLC1* (At5g58670) is induced by dehydration and salt stress [7,8]. *AtPLC3* (At4g38530) is required for the secondary response to abscisic acid signaling [9], and *AtPLC9* (At3g47220), together with *AtPLC3*, is involved in thermotolerance [10,11]. On the other hand, *AtPLC2* (At3g08510) is constitutively expressed even under environmental stresses [7,12]. Although much progress has been made in understanding of involvement of *AtPLC* genes in environmental stress responses, we still do not know which of the nine *AtPLC* isoforms plays a major role in phosphoinositide metabolism.

At the cellular level, environmental stimuli such as salt and heat stresses induce ER stresses [13,14], which are caused by accumulation of aberrant proteins in the ER. To cope with the ER stress, eukaryotic cells have evolved multiple conserved strategies, termed ER quality control. The ER quality control system consists of the unfolded protein response (UPR) [15–17], ER-associated degradation (ERAD) [18–20], and the translational attenuation [21]. The system monitors the folding status of newly synthesized proteins in the secretory pathway, thereby allowing only correctly folded proteins to advance from the ER to their final destinations [22]. When aberrant proteins are accumulated in the ER, cells activate the UPR pathway to recover folding processes by increasing folding capacity. Concurrently, cells are able to suppress protein translation to reduce the loading of newly synthesized proteins into the ER [21]. Nevertheless, ultimately misfolded proteins are sorted and degraded to diminish their potential toxicity via the ERAD pathway [19,20]. Recent studies of ER quality control in plants showed that the underlying mechanisms are highly similar to those in mammals and yeasts in terms of UPR and ERAD pathways [23–26]. Environmental stresses such as salt or heat up-regulate most of *AtPLC* genes expression [7], and these stresses concurrently generate the ER stress in Arabidopsis [25]. However, a connection between phosphoinositide signaling and ER stress response remains unknown. Given that the ER is the major site of phospholipid biosynthesis, the phospholipid signaling may well be associated with ER function [27]. Thus, involvement of lipid signaling in ER stress response is an emerging issue in plants.

In this study, we isolated and analyzed knockout mutants of multiple AtPLC isoforms in Arabidopsis. Lipidomic profiling of phosphoinositides in the mutants showed that AtPLC2 is a primary PI-PLC in phosphoinositide metabolism. AtPLC2 protein is highly expressed in trichomes of young rosettes, young floral buds and vascular tissues of leaves, petals and roots. Subcellular localization study showed that AtPLC2 is found in the plasma membranes in different cell types. A knockout mutant *plc2-1* showed seedling growth defects. Furthermore, the *plc2-1* mutant showed enhanced susceptibility to stress affecting ER homeostasis. Thus, AtPLC2 is a major PI-PLC affecting phosphoinositide levels and ER stress tolerance.

## Results

### Isolation of PI-PLC mutants in Arabidopsis

To determine the primary AtPLC isoform in phosphoinositide metabolism, we first attempted to obtain T-DNA mutants for nine AtPLC isoforms and analyzed their phosphoinositide profiles. Although mutants for *AtPLC5* (At5g58690), *AtPLC7* (At3g55940), *AtPLC8* (At3g47290) and *AtPLC9* (At3g47220) were not available from public seed libraries, homozygous mutants were isolated for the other isoforms. Nomenclature of *AtPLC* genes follows those defined by Mueller-Roeber and Pical (2002) and Hunt *et al*. (2004) [5,6]. The positions of T-DNA insertion were confirmed by PCR-based sequencing, which were all located within the protein coding sequence of relevant genes (Fig 1B). The full-length transcript was not detected in any of the mutants, which confirmed that they are all null mutants (Fig 1C). Thus, the single mutants of these PI-PLC isoforms are viable.

### Differential gene expression pattern of *AtPLC* genes

**Fig 2:**
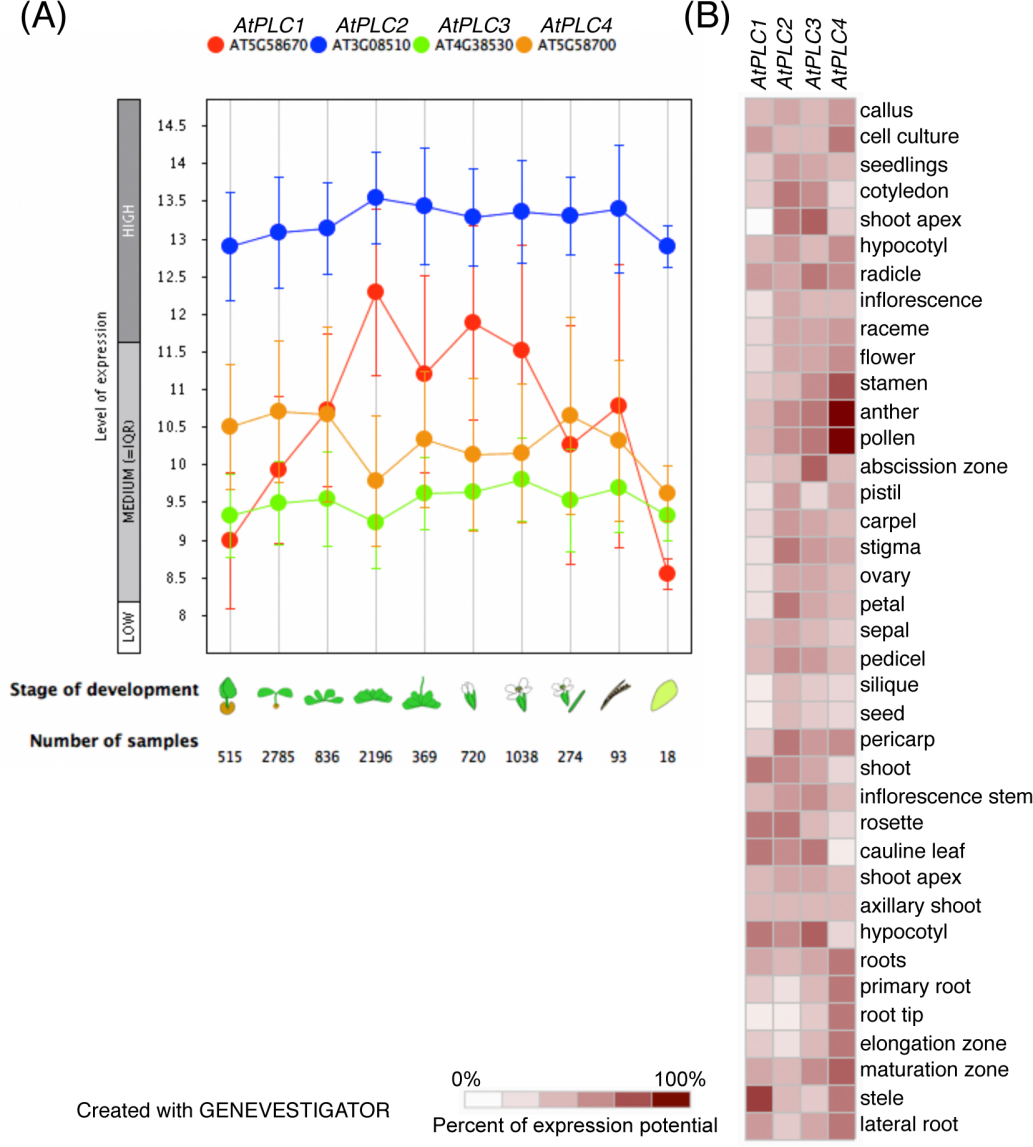
Gene expression patterns of *AtPLC1*, *AtPLC2*, *AtPLC3*, and *AtPLC4*. (A) Developmental stage-specific expression patterns of *AtPLC1*, *AtPLC2*, *AtPLC3*, and *AtPLC4*. Left to right, germinating seed, seedling, young rosette, developed rosette, bolting rosette, young flower, developed flower, flower and silique, mature silique, senescence. “HIGH”, “MEDIUM”, and “LOW” expression were calculated by microarray assay. The number of samples indicates microarray gene expression data collected by GENEVESTIGATOR (www.genevestigator.com). (B) Heat map of tissue-specific expression pattern of *AtPLC1*, *AtPLC2*, *AtPLC3*, and *AtPLC4*. Data were analyzed with GENEVESTIGATOR.

To examine differential gene expression profiles of *AtPLC* genes whose mutants were obtained in Fig 1, we utilized the publicly available gene expression database (GENEVESTIGATOR; www.genevestigator.com). Data for *AtPLC6* were not available. We found differential expression patterns of these four *AtPLC* genes during plant growth and development (Fig 2A); *AtPLC2* had the highest expression level of the four isoforms consistently throughout the development. While *AtPLC3* and *AtPLC4* showed stable expression pattern as well, *AtPLC1* expression was increased during vegetative growth, remained high during flower development but was dramatically decreased along with senescence. Regarding tissue-specificity, *AtPLC2* expressed rather ubiquitously in most of tissues except roots including primary root and root tip (Fig 2B). However, the other isoforms have clearer tissue specificity; *AtPLC1* at stele, *AtPLC3* at the shoot apex, abscission zone and hypocotyl, and *AtPLC4* in male reproductive parts of flowers. These data suggest that *AtPLC2* is the primarily and ubiquitously expressed isoform.

### Altered phosphoinositide content in the *plc2-1* mutant

**Fig 3:**
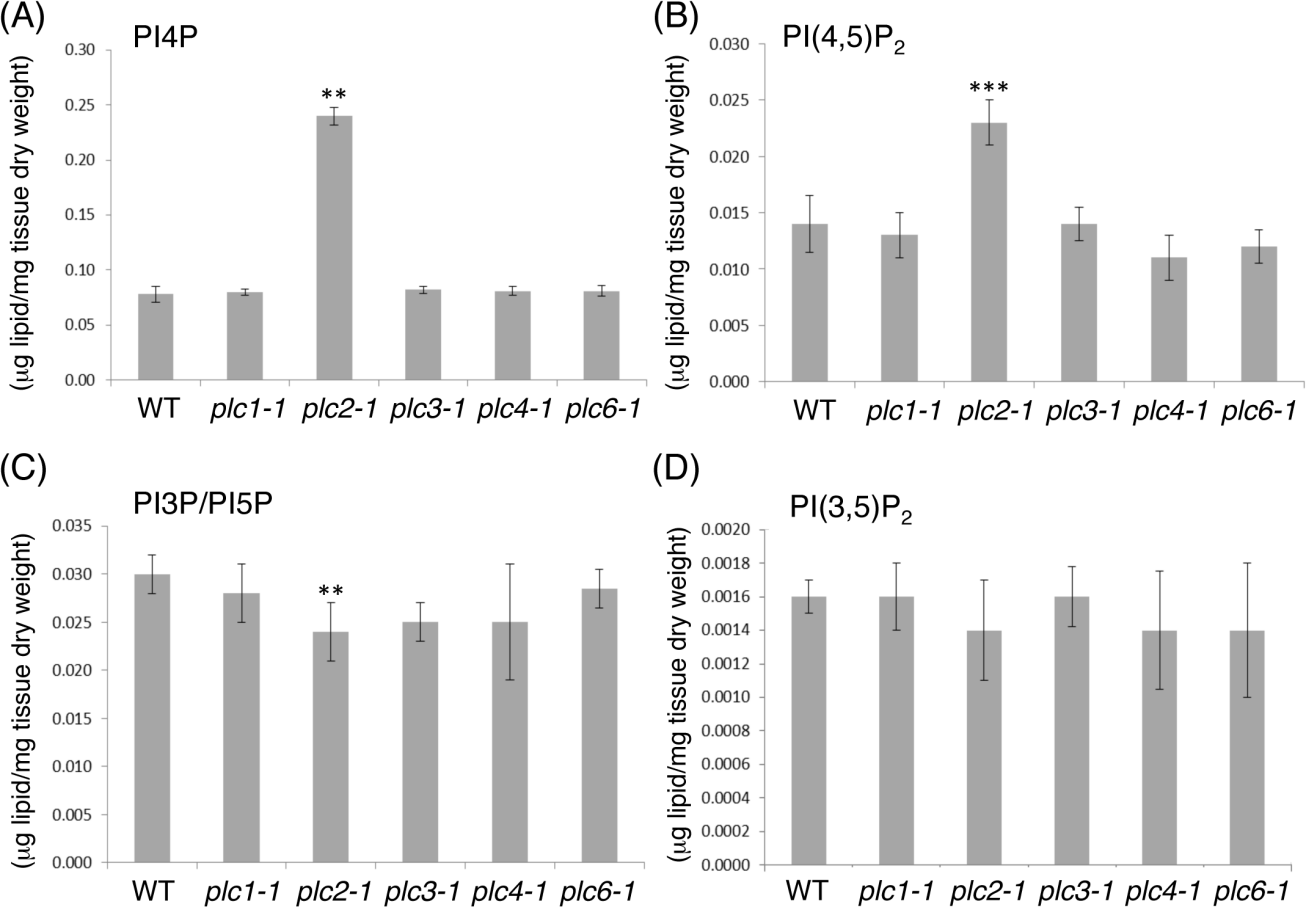
Lipidomic profiling of phosphoinositide levels in 14-day-old seedlings of the *plc1-1*, *plc2-1*, *plc3-1*, *plc4-1*, *plc6-1* mutants compared with the wild type (WT). (A) Phosphatidylinositol 4-phosphate (PI4P), (B) phosphatidylinositol 4,5-bisphosphate [PI(4,5)P_2_], (C) sum of phosphatidylinositol 3-phosphate (PI3P) and phosphatidylinositol 5-phosphate (PI5P), (D) phosphatidylinositol 3,5-bisphosphate [PI(3,5)P_2_]. Data are mean±SD of 4 biological replicates. **P<0.01, ***P<0.001 (by Student’s *t*-test).

To investigate which AtPLC isoform has major contribution to the phosphoinositide metabolism, we performed lipidomic analysis and quantified the phosphoinositide levels of the AtPLC mutants (Fig 3). Among the five AtPLC mutants isolated, only the *plc2-1* mutant showed a 2.5-fold increase in level of phosphatidylinositol 4-phosphate (PI4P) (Fig 3A) and a 1.8-fold increase in that of PI(4,5)P_2_ (Fig 3B). The sum of phosphatidylinositol 3-phosphate (PI3P) and phosphatidylinositol 5-phosphate (PI5P) levels showed a slight but significant decrease in the *plc2-1* mutant (Fig 3C). However, phosphatidylinositol 3,5-bisphosphate [PI(3,5)P_2_] levels (Fig 3D) were not altered in any of these mutants. Because PI4P is a precursor of PI(4,5)P_2_, an increase in levels of both PI(4,5)P_2_ and PI4P suggests that a major pathway of PI(4,5)P_2_ hydrolysis is impaired in the *plc2-1* mutant.

### Tissue localization of AtPLC2

**Fig 4:**
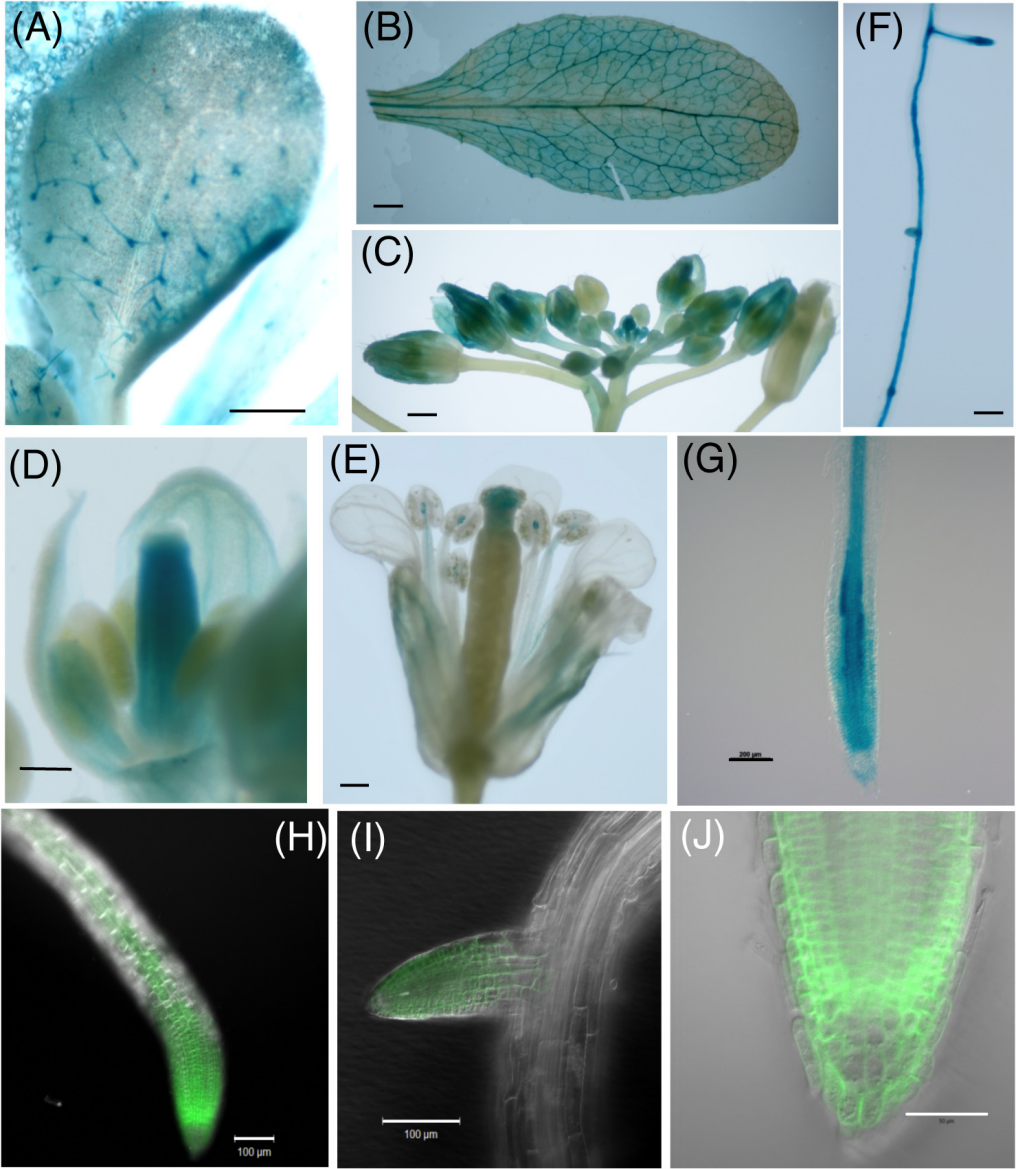
Tissue expression of AtPLC2. (A-G) Tissue expression of AtPLC2 by histochemical GUS staining of *ProPLC2*:*PLC2-GUS* transgenic plants. (A) Developing rosette leaf of a 7-day-old seedling, (B) developed rosette leaf of a 3-week-old plant, (C) inflorescence with floral buds in different developmental stages, (D) floral bud with developing reproductive organs, (E) mature flower, (F) part of the main root of a 2-week-old seedling and (G) tip of the main root of a 2-week-old seedling. (H-J) Localization of fluorescent PLC2-Venus in roots of 2-week-old seedlings of *ProPLC2*:*PLC2-Venus* transgenic plants. (H) Main root, (I) emerging lateral root branch, (J) tip of the main root. Scale bars are 500 μm in (A) to (C), 200 μm in (D) to (G), 100 μm in (H) and (I) and 50 μm in (J).

*AtPLC2* transcript is ubiquitously detected in most of plant tissues as shown in Fig 2B [7,12]; however, little is known about the protein localization of AtPLC2. To explore the tissue localization of AtPLC2 protein, we created a transgenic plant that expresses *AtPLC2* fused to a *GUS* gene driven by the *AtPLC2* promoter (*ProPLC2*:*PLC2-GUS*). GUS staining was observed in trichomes, particularly at the base, in developing true leaves of *ProPLC2*:*PLC2-GUS* transgenic plants (Fig 4A). As the leaves developed, staining was found in leaf vasculature but not at the trichome (Fig 4B). In the inflorescences, staining was found in young buds; entire buds were stained in the early stages (Fig 4C), while carpels and vasculature of petals were stained after the onset of reproductive organ development (Fig 4D). In mature flowers, staining was restricted to the stigma, filament and the boundary region between the filament and stamen (Fig 4E). In roots, GUS staining was found in vasculature (Fig 4F). In addition, intense staining was observed at the branch of lateral roots and root tips. A closer look at the tip of a primary root showed differential patterns of GUS staining: intense and uniform distribution at cell division and elongation zones but restricted staining at vasculature in maturation zones (Fig 4G).

### Subcellular localization of AtPLC2 protein

Next, to observe the subcellular localization of AtPLC2 protein, we created a transgenic line that expresses *AtPLC2* fused to the fluorescent protein Venus (*ProPLC2*:*PLC2-Venus*). In agreement with GUS staining, strong signals appeared at the tip of main roots (Fig 4H) and branching lateral root (Fig 4I). At the root tips, fluorescent signals were more evenly distributed across different cell types except columellae (Fig 4J). This localization is in agreement with previous biochemical study of anti-PLC2 antibody detecting enrichment of the antigen in the plasma-membrane-enriched fraction [28].

**Fig 5:**
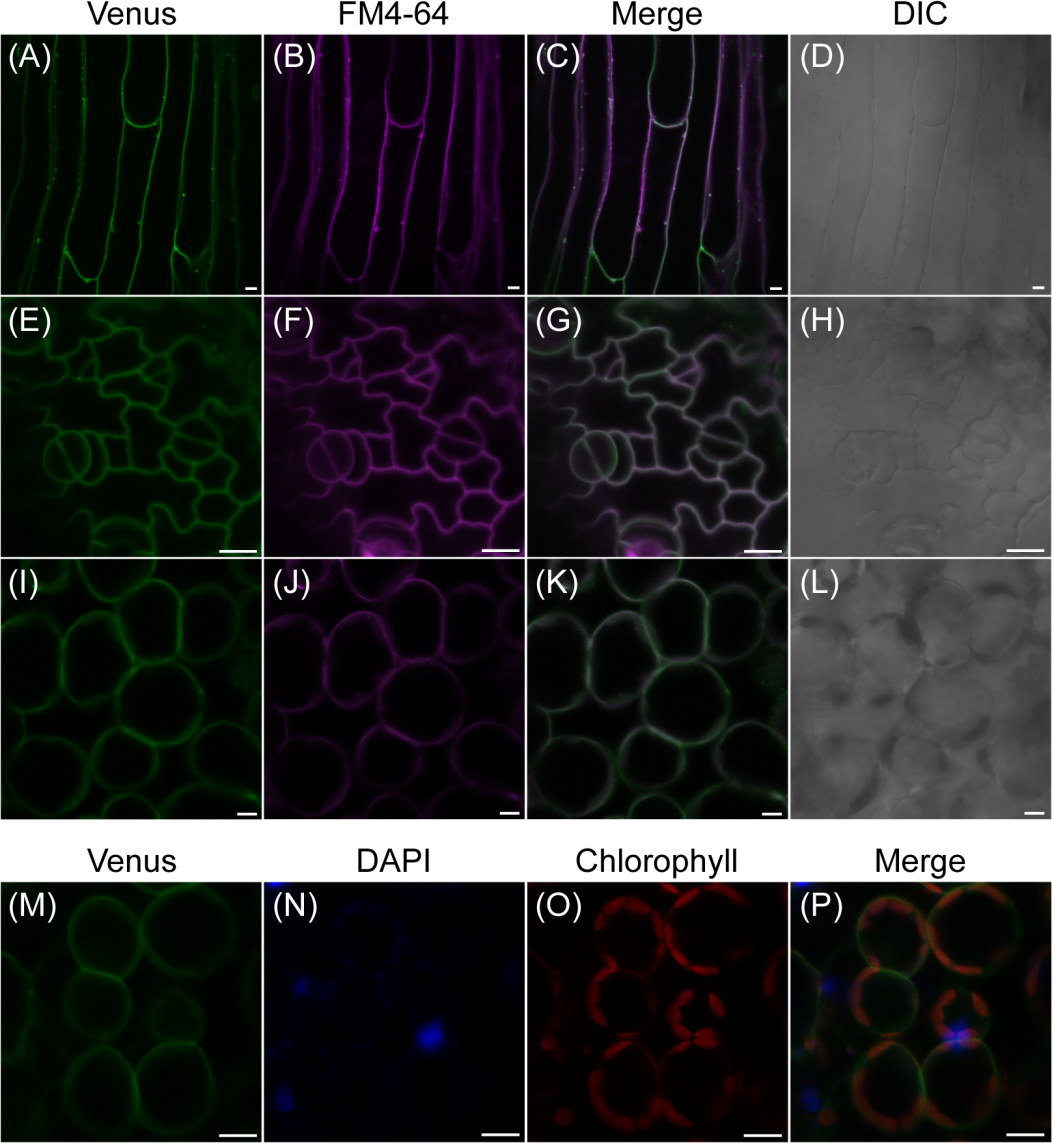
Subcellular localization of fluorescent AtPLC2-Venus in 12-day-old seedlings of *ProPLC2*:*PLC2-Venus* transgenic plants. (A-D) Fluorescence of ProPLC2:PLC2-Venus (A) and staining of plasma membranes by FM4-64 dye (B) were merged (C) at stem epidermis. (E-H) Fluorescence of ProPLC2:PLC2-Venus (E) and staining of plasma membranes by FM4-64 dye (F) were merged (G) at leaf pavement and guard cells. (I-L) Fluorescence of ProPLC2:PLC2-Venus (I) and staining of plasma membranes by FM4-64 dye (J) were merged (K) at leaf mesophyll cells. (D), (H), and (L) are differential interference contrast (DIC) images for each sample. (M-P) Fluorescence of ProPLC2:PLC2-Venus (M), staining of nuclei by DAPI (N) and chlorophyll autofluorescence (O) were merged (P) at leaf mesophyll cells. Scale bars are 10 μm.

To further study the subcellular localization of AtPLC2 in different tissues, we observed stem epidermal cells (Fig 5A–5D), leaf epidermal cells (Fig 5E–5H), and mesophyll cells (Fig 5I–5L). In all cells observed here, the Venus signals were co-localized with the plasma membrane marker FM4-64 but not with the nuclear marker 4’,6’-diamino-2’-phenylindole (DAPI) or autofluorescence of chloroplasts (Fig 5M–5P). In addition to supporting the previous study [28], these observations suggest that AtPLC2 localizes mainly at the plasma membrane in different cell types.

### Seedling growth retardation of the *plc2-1* mutant

**Fig 6:**
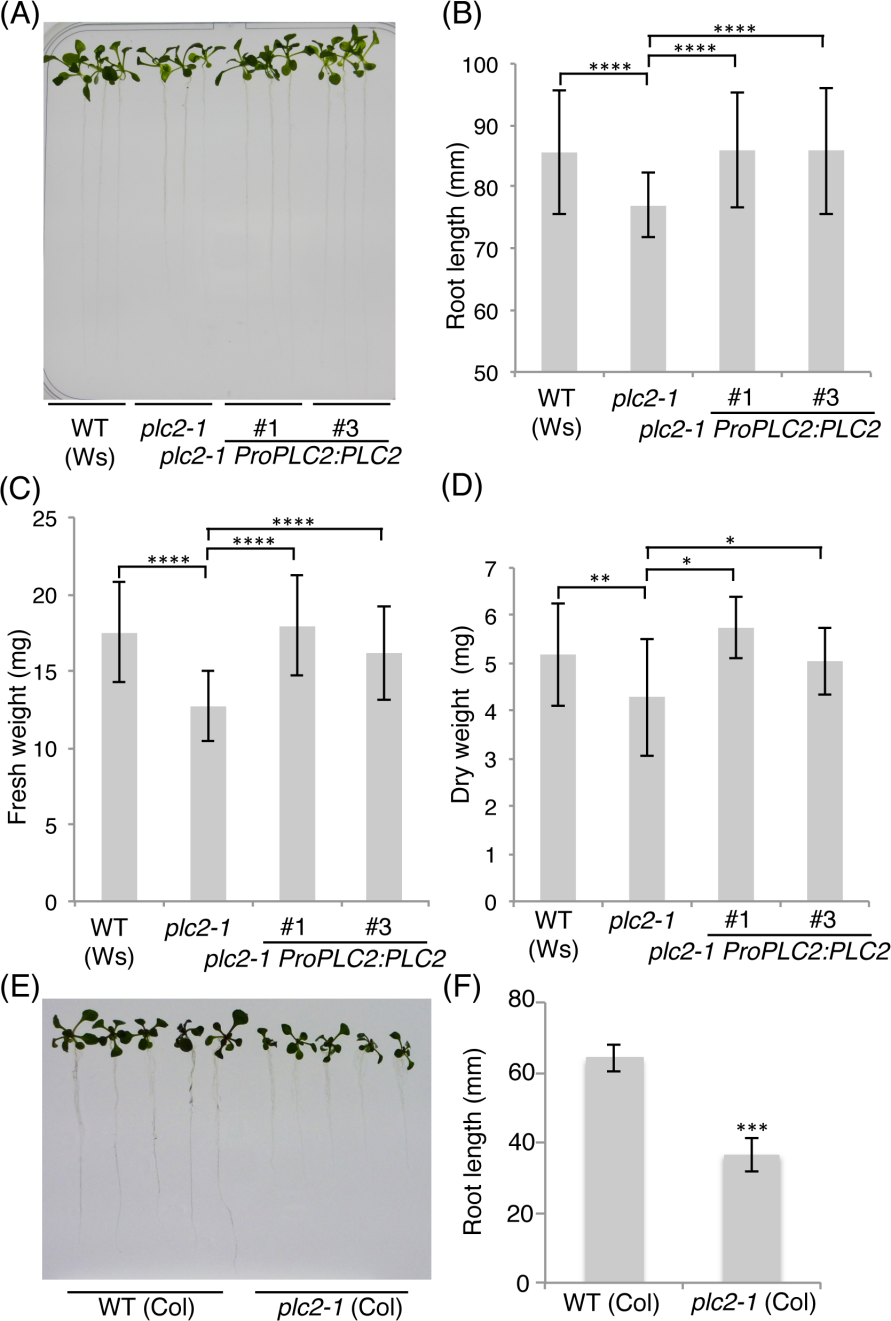
Seedling growth defect of the *plc2-1* mutant. (A) The wild-type (Ws), *plc2-1* and two independent *plc2-1 ProPLC2*:*PLC2* transgenic plants (#1 and #3) were grown for 15 days on MS media, and representative plants were photographed. (B) Root length of plants shown in (A). (C) Fresh weight of a plant shown in (A). (D) Dry weight of 5 plants shown in (A). In (B), (C), and (D), data are mean±SD of 30 seedlings and three biologically independent experiments were performed with similar results. *P<0.05, **P<0.01, ****P<0.0001 (Student’s *t*-test). (E) The wild-type and *plc2-1* (Col) plants were grown for 15 days on MS media, and representative plants were photographed. (F) Root length of plants shown in (E). Data are mean±SD of 16 seedlings and three biologically independent experiments were performed with similar results. ***P<0.001 (Student’s *t*-test).

**Fig 7:**
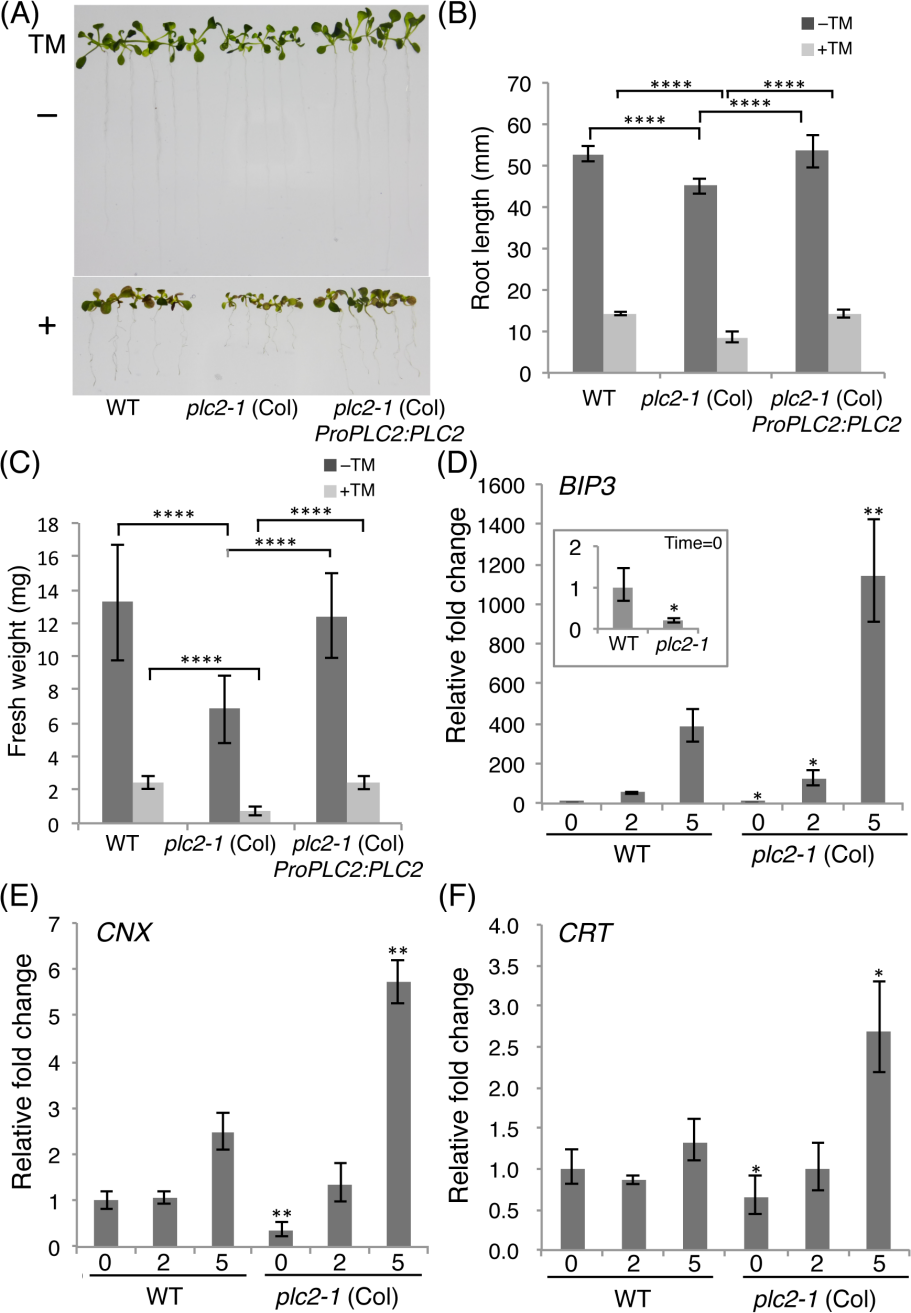
Enhanced susceptibility of the *plc2-1* mutant to the ER stress. (A) The wild type, *plc2-1* (Col) and *plc2-1* (Col) *ProPLC2*:*PLC2* (line #33) were grown on MS media for 7 days, and were grown on MS media containing 0.3 μg/ml of tunicamycin (TM) for additional 6 days. The representative seedlings were photographed on 13^th^ day (the lower panel). The seedlings without TM treatment were shown in the upper panel as control. (B) Root length measurement of (A). (C) Fresh weight of a plant shown in (A). In (B) and (C), data are mean±SD of 30 seedlings and three biologically independent experiments were performed with similar results. ****P<0.0001 (Student’s *t*-test). (D) qRT-PCR analysis of *binding protein 3* (*BiP3*) transcripts in the wild-type and the *plc2-1* (Col) plants in response to the ER stress. RNA was extracted from the 7-day-old plants treated with 5 μg/ml TM at each time point (0, 2, 5 hours of the treatment). The expression level of the wild type at the time zero was set to 1. Data at time 0 were magnified at the inset panel. (E) qRT-PCR analysis of *calnexin* (*CNX*) transcripts in the wild-type and the *plc2-1* (Col) plants in response to the ER stress as described in (D). (F) qRT-PCR analysis of *calreticulin* (*CRT*) transcripts in the wild-type and the *plc2-1* (Col) plants in response to the ER stress as described in (D). In (D), (E), and (F), Student’s *t*-test was performed between the wild-type and *plc2-1* (Col) plants at each time point. *P<0.05, **P<0.01, ****P<0.0001.

The seedlings of *plc2-1* cultured on solid Murashige and Skoog (MS) media showed growth retardation both in shoots and roots (Fig 6A). The overall root length of 15-day-old seedlings was significantly reduced as compared with that of the wild-type plants in Wassilewskija (Ws) ecotype (Fig 6B). The fresh weight and dry weight were also reduced by nearly 70% and 80%, respectively, compared with that of the wild-type plants (Fig 6C and 6D). These growth retardations of the *plc2-1* mutant were fully complemented in two independent lines harboring a genomic sequence of *AtPLC2* (*ProPLC2*:*PLC2*) in the *plc2-1* mutant (*plc2-1 ProPLC2*:*PLC2* #1 and #3) in root length, fresh weight and dry weight (Fig 6A–6D). To exclude a possibility that this growth defect is ecotype-specific, the *plc2-1* mutant was introgressed into Columbia-0 (Col) ecotype by backcrossing six times. In consistent with that in the Ws background, the *plc2-1* (Col) mutant showed growth retardation both in shoots and roots (Fig 6E and 6F). Moreover, this growth defect was also fully complemented by heterologous expression of *AtPLC2* in the *plc2-1* (Col) [*plc2-1* (Col) *ProPLC2*:*PLC2*] as shown in Fig 7A–7C. These observations indicate that the growth defect in the *plc2-1* mutant is due to knocking out of *AtPLC2*.

### Enhanced susceptibility of the *plc2-1* roots to ER stress

To investigate whether phosphoinositides are involved in ER stress tolerance, we examined sensitivity of the *plc2-1* mutant against the tunicamycin (TM)–induced ER stress. TM inhibits the protein *N*-glycosylation pathway in the ER and thus induces the ER stress. Treatment of TM produced growth defects in the wild-type seedlings: smaller rosette leaves and shorter roots (Fig 7A). These phenotypes were even stronger in the *plc2-1* mutant (Fig 7A). In response to the ER stress, root length of the *plc2-1* mutant was shortened to 19.1% that of control treatment, whereas that of the wild type was shortened by 27.3% (Fig 7B). Consistent with this, the fresh weight of whole seedlings of the *plc2-1* mutant was reduced to 10.8% that of control treatment, whereas that of the wild type was reduced to 18.4% in response to the ER stress. These defects in the *plc2-1* mutant were complemented in the *plc2-1* (Col) *ProPLC2*:*PLC2* transgenic plant: reductions of root length and fresh weight upon the ER stress were 26.7% and 19.9%, respectively (Fig 7B and 7C). To determine whether this enhanced growth defect was due to a compromised ER stress response, we examined the expression of ER stress responsive genes [29]: *binding protein 3* (*BiP3*), *calnexin* (*CNX*) and *calreticulin* (*CRT*) [30,31]. Expression of all of these genes was significantly higher in the *plc2-1* mutant than in the wild type (Fig 7D–7F) in response to the ER stress. In addition, gene expression levels at 0 h were lower in the *plc2-1* mutant than in the wild type (Fig 7D–7F). These data suggest that the *plc2-1* mutant is hypersensitive to the ER stress. Thus, AtPLC2 function contributes to the ER stress response.

## Discussion

The Arabidopsis *AtPLC2* was first cloned by Hirayama *et al*. (1997) after the first cloning and functional analysis of *AtPLC1* [8]. AtPLC2 shows PI(4,5)P_2_-specific PLC activity *in vitro* and requires 1–100 μM Ca^2+^ for its activity [28]. However, because of lack of *AtPLC2* mutant analyses, the *in vivo* role of AtPLC2 in the entire phosphoinositide metabolism was unclear. Given that at least nine AtPLCs have been reported in Arabidopsis [5,6], whether any of these have a decisive role in phosphoinositide metabolism or whether most have redundant roles in the process is a major issue to be addressed [1]. Here, we isolated AtPLC mutants and profiled their phosphoinositide levels (Fig 3). Knockout of *AtPLC2* significantly increased levels of PI(4,5)P_2_, a primary substrate of PI-PLC, suggesting that AtPLC2 may be a major PI-PLC involved in phosphoinositide metabolism.

We found no significant changes in phosphoinositide levels in the *plc1-1*, *plc3-1*, *plc4-1*, and *plc6-1* mutants, even though corresponding genes *AtPLC1*, *AtPLC3*, *AtPLC4*, and *AtPLC6* encode functional proteins [8,9,32]. These *PI-PLC* genes are transcriptionally induced by various stresses such as salt, cold and dehydration, whereas *AtPLC2* expression is stable but higher than that of the others (Fig 2A) [7,12]. These AtPLCs may be conditional isoforms contributing to specific conditions with specific localization, whereas *AtPLC2* expresses continuously and serves for the basal metabolism of phosphoinositides, thus affecting their levels when knocked out. It would be of interest to determine whether different PLCs affect phosphoinositide levels differently in specific tissues or under different stress conditions in future studies.

Our reporter assay by GUS and Venus revealed that AtPLC2 is primarily expressed in vascular tissues in leaves (Fig 4B), petals (Fig 4D), filaments (Fig 4E), and roots (Fig 4F and 4G). Moreover, AtPLC2 expression was observed in tissues with active cell proliferation, such as trichomes (Fig 4A), young floral buds (Fig 4C), and root founder cells (Fig 4F). Previously, GUS reporter assay was conducted for AtPLC1, AtPLC4 and AtPLC5 [6], which showed that both AtPLC1 and AtPLC5 were expressed in vascular tissues of roots and leaves but AtPLC4 was expressed exclusively in pollen and in cells of the stigma surface. Besides, AtPLC5 was also expressed in guard cells and various floral organs. Based on our observation, while expression of AtPLC2 in vascular tissues was similar to that of AtPLC1 and AtPLC5, expression of AtPLC2 in tissues with active cell proliferation was not observed in the previously studied PLC isoforms and thus was a distinct feature. This tissue specificity corresponds with the suggested roles of phosphoinositides. The contribution of phosphoinositides in vascular tissues is suggested by analysis of mutants affecting phosphoinositide phosphatases. *FRAGILE FIBER 3* encodes inositol polyphosphate 5-phosphatase and is required for secondary wall synthesis and actin organization in fiber cells [33]. A specific PI(3,5)P_2_ phosphatase encoded by *SAC1* is involved in cell morphogenesis, cell wall synthesis and actin organization [34]. *VAN3*, which is essential in leaf vascular development, has a pleckstrin homology domain that binds PI4P [35]. Indeed, CVP2, an inositol 5-phosphatase whose knockout confers a phenotype similar to that of *van3*, acts partially with VAN3 to regulate the continuity of vasculature [36,37]. In line with this evidence which suggests an involvement of multiple phosphoinositide phosphatases in vascular development, altered levels of PI(4,5)P_2_ or IP_3_ by AtPLC2 may influence the substrate availability of these phosphatases, thus affecting seedling growth.

The seedlings of *plc2-1* were smaller than those of the wild type in both Ws and Col ecotypes (Figs 6 and 7). The growth defect was complemented by transforming *ProPLC2*:*PLC2*, which indicates that AtPLC2 is the primary isoform that contributes to seedling growth. Reduced primary root length of the *plc2-1* mutant suggests reduced competence of the root apical meristem. PI4K is important in trafficking from the trans-Golgi network to the prevacuolar compartment [38]. AtPLC2 may cooperate with PI4K for a straightforward signaling pathway for efficient generation of IP_3_ and DAG.

The *plc2-1* mutant showed hypersensitivity against the TM-induced ER stress. Although there is still limited information about the connection between phosphoinositides and ER homeostasis, membrane stress caused by imbalanced lipid composition triggers the UPR to reprogram protein homeostasis in yeast [39]. In addition, inhibition of PC biosynthesis in mammalian cells induces ER stress presumably by significant alteration of membrane lipid composition [40]. Furthermore, phosphoinositides are involved in vesicular membrane trafficking [38]. In Arabidopsis, microarray analysis showed ER stress-induced expression of gene encoding PI4Kγ2, which may catalyze a reaction to produce PI4P and provide a substrate for AtPLCs (Fig 1A) [23]. Altered phosphoinositide composition will affect membrane integrity or vesicle trafficking, causing a hypersensitive response to the ER stress.

In animal and microbial research fields, function of PLCs has been studied extensively. However, involvement of PLCs in ER stress is not well-known. For example, ER stress decreases the expression of PLC-β1 isoform in neurons [41]. Regarding the connection between phosphoinositides and ER homeostasis, involvement of phosphatidylinositol 3-kinase (PI3K) in ER stress response has been suggested. For example, suppression of p85α, a regulatory subunit of class Ia PI3K reduces ER stress-dependent accumulation of nuclear X-box-binding protein-1 (XBP-1), a transcriptional regulator of the UPR [42,43]. This decreases induction of UPR target genes and stimulates apoptosis [43]. Moreover, involvement of phosphatidylinositol synthesis in ER stress is reported in Zebrafish [44,45]. These studies highlight an importance of entire phosphoinositide signaling in ER stress response. Our finding that knocking out of AtPLC2 altered phosphoinositide profiles and caused hypersensitivity to ER stress in Arabidopsis not only supports the importance of phosphoinositides but highlights a crucial role of PLC function in ER stress, which is not described clearly in the animal research field. Plants may possess a distinct regulatory mechanism to transduce phosphoinositide signals under the ER stress.

In consistent with the previous study [28], AtPLC2 mainly localized at the plasma membrane (Fig 5). It is intriguing that plasma membrane-localized AtPLC2 is involved in the ER stress response. Recently, Yang *et al*. (2014) reported that a plasma membrane-associated transcription factor, NAC062, mediates the UPR in Arabidopsis [46]. NAC062 relocates from the plasma membrane to the nucleus upon the ER stress, and then regulates gene expression downstream of UPR. NAC062 is one of the plant-specific NAC (NAM, ATAF1,2, CUC2) transcription factors, and its expression is up-regulated by the ER stress. However, how the plasma membrane-associated NAC062 responds to the ER stress is poorly understood. Interestingly, a highly dynamic organelle was observed that is derived from the ER and serves as a major source of phosphatidylinositol to supply phosphoinositides to cellular membranes [47]. It is possible that plasma membrane-localized AtPLC2 might also take ER-derived phosphoinositides as a substrate and play a role in the ER stress response. Furthermore, a direct physical connection has been observed between the plasma membrane and the ER [48]. It is possible that AtPLC2-generated signals may be transduced to the ER through the direct contact site. Higher plants may have evolved a unique mechanism involving cross-talk between the ER and plasma membrane to cope with the ER stress.

In conclusion, AtPLC2 is the major PI-PLC involved in phosphoinositide metabolism and seedling growth in Arabidopsis. Moreover, it is important for the ER stress tolerance, which suggests the link between phosphoinositide signaling and the ER stress response. We suggest AtPLC2 as a key enzyme in the phosphoinositide metabolic system and a novel concept in ER homeostasis associated with phosphoinositides evidenced by the functional study of the *plc2-1* mutant.

## Methods

### Plant materials and growth condition

Arabidopsis plants (Columbia-0 ecotype, unless specified) were grown under 16-h light/8-h dark condition at 22°C. Murashige and Skoog (MS) media were used at ½ concentration for plant culture [49]. Mutant seeds were obtained from The Arabidopsis Information Resource (TAIR), except for *plc2-1* seeds, which were obtained from the Institut National de la Recherche Agronomique (INRA): *plc1-1* (SALK_025769), *plc2-1* (FLAG_506C04), *plc3-1* (CS879873), *plc4-1* (CS876876), *plc6-1* (SALK_041365). The *plc2-1* mutant was in Wassilewskija (Ws) ecotype, and was therefore backcrossed with Col-0 ecotype six times to replace the ecotypical background. The obtained mutant was named *plc2-1* (Col).

### Plasmid vector construction and plant transformation

For genomic complementation of the *plc2-1* mutant, about 4 kb of the genomic sequence for *AtPLC2* was amplified by PCR with oligonucleotide primers KY101 and KY102 and cloned into the pENTR/D-TOPO plasmid vector (Invitrogen, Carlsbad, CA) to obtain pYN2030. Positive clones were sequenced to confirm accuracy and were recombined to the pHGW destination vector by use of LR clonase (Invitrogen, Carlsbad, CA) [50]. The obtained plasmid, pYN3051, was transformed into Agrobacterium GV3101 for transformation into the *plc2-1* and *plc2-1* (Col) mutants.

The GUS reporter construct was created as follows: the *Sfo*I site was introduced immediately before the stop codon of pYN2030 by site-directed mutagenesis [51] with the oligonucleotide primer KY103 to obtain pYN2031. Next, a GUS fragment was inserted into the *Sfo*I site to produce pYN2036. This entry vector was recombined to the pHGW destination vector by use of LR clonase. The resulting plasmid pYN3045 was transformed into Agrobacterium GV3101 for transformation into the *plc2-1* mutant.

The Venus fluorescent reporter construct was created by inserting a triple Venus fragment into pYN2031. The obtained entry vector pYN2037 was recombined to the pHGW destination vector by use of LR clonase and the resulting plasmid pYN3037 was transformed into Agrobacterium GV3101 for transformation into the *plc2-1* mutant. All transformed plants were screened on MS media containing hygromycin.

### Lipid extraction and profiling

Lipid extraction was conducted under the acidic condition for efficient recovery of phosphoinositides as described [52]. Briefly, samples were ground in 10 volumes of extraction solvent (ice-cold chloroform:methanol:concentrated HCl = 100:100:0.7) and centrifuged, then supernatant was separated. The remaining pellet was re-extracted with 4 volumes of extraction solvent and centrifuged to recover the supernatant. Two supernatant fractions were combined with 2 volumes of 0.6 N HCl, mixed, centrifuged, and the upper phase was removed. The lower phase was washed 3 times with 4 volumes of washing solvent (chloroform:methanol:0.6 N HCl = 3:48:47), dried and dissolved in chloroform:methanol = 2:1 for storage at -80°C.

Analysis of PI4P, PI3P/PI5P, PI(4,5)P_2_, and PI(3,5)P_2_ levels was quantified as described [53] by use of Dionex Ion Chromatography 3000 (Dionex, Sunnyvale, CA, USA). Lipid extracts were deacylated by incubation with 0.5 ml methylamine reagent [MeOH/40% methylamine in water/1-butanol/water (47:36:9:8)] at 50°C for 45 min. The aqueous phase was dried, resuspended in 0.5 ml of 1-butanol/petroleum ether/ethyl formate (20:40:1) and extracted twice with an equal volume of water. Aqueous extracts were dried, resuspended in water, and subjected to anion-exchange high-performance liquid chromatography on an Ionpac AS11-HC column (Dionex, Sunnyvale, CA, USA). Negatively charged glycerol head groups were eluted with a 1.5–86 mM KOH gradient and detected online by suppressed conductivity 75 in a Dionex ion chromatography system equipped with an ASRS-ultra II self-regenerating suppressor. Individual peaks were identified, and peak areas were calculated by use of Chromeleon (Dionex, Sunnyvale, CA, USA). Lipid levels were calculated with deacylated anionic phospholipids using as standards.

### GUS staining

Harvested fresh tissues other than roots were immediately immersed in ice-cold 90% (v/v) acetone for 15 min, then with GUS staining solution [10 mM EDTA, 5 mM potassium ferricyanide, 5 mM potassium ferrocyanide, 0.1% (w/v) Triton X-100, 0.5 mg/ml X-Gluc (5-bromo-4-chloro-3-indolyl-β-_D_-glucuronide) in 100 mM phosphate buffer]. After incubation in the dark at 37°C for 30 min, staining was stopped by replacing the solution with 70% ethanol. For colored tissues, pigments were removed by immersing the tissue in ethanol:acetic acid = 6:1 (in vol).

### Tunicamycin treatment

For phenotypical observations, the plants were grown on MS media for 7 days, and then transferred to MS media containing 0.3 μg/ml tunicamycin for an additional 6 days of culture. For the quantitative RT-PCR (qRT-PCR) analysis, the plants were grown on MS media for 7 days. Then, the seedlings were transferred to MS media containing 5 μg/ml tunicamycin, and were harvested at the times indicated as described [23]. Dimethyl sulfoxide (DMSO) was used as a control for both experiments.

### Confocal laser-scanning microscopy

Venus expression in the *ProPLC2*:*PLC2-Venus* transgenic line in 12-day-old seedlings was observed under a microscope (LSM 510 Meta; Carl Zeiss, Jena, Germany) equipped with LCI Plan-Neofluar 63x/1.3 NA immersion, Plan-Apochromat 20x/0.8 NA, and Plan-Apochromat 10x/0.45 NA. For plasma membrane staining, seedlings were immersed in 10 μg/ml of FM 4–64 (Invitrogen, Carlsbad, CA, USA) for 10 min and subjected to confocal microscopy observation. For nucleus staining, samples were immersed in 10 μg/ml of DAPI (Invitrogen, Carlsbad, CA, USA) for 10 min and subjected to confocal microscopy observation. Images were captured by use of LSM 510 v3.2 (Carl Zeiss, Jena, Germany) with filters for Venus (514 nm laser, BP 520–555), FM 4–64 (543 nm laser, BP 560–615), DAPI (405 nm laser, BP 420–480), and chlorophyII autofluorescence (488 nm laser, LP 650).

### RT-PCR analysis

Total RNA was isolated from 2-week-old seedlings except *AtPLC6* using plant RNeasy mini kit (Qiagen, Dusseldorf, Germany) according to the manufacture’s instruction. RNase-free DNase (Qiagen, Dusseldorf, Germany) was used during the on-column digestion to remove genomic DNA contamination. For *AtPLC6*, total RNA isolated from the flowers of 5-week-old plants were used because *AtPLC6* highly expresses during flower development [54]. The cDNA was synthesized by the SuperScriptIII First-Strand Synthesis System (Invitrogen, Carlsbad, CA, USA) and used as the templates for RT-PCR analysis. *Actin* (*ACT*) was used as a control. The primers used are listed in S1 Table.

### Quantitative RT-PCR (qRT-PCR)

Total RNA was isolated from 7-day-old seedlings using TRI reagent (Ambion, Austin, TX, USA) including DNase treatment according to the manufacture’s instruction, and cDNA was synthesized by the SuperScriptIII First-Strand Synthesis System (Invitrogen, Carlsbad, CA, USA). qRT-PCR was performed with the 7500 Real Time PCR System (Applied Biosystems, Foster city, CA, USA). The comparative threshold cycle (CT) method was used to determine the relative amount of gene expression, with the expression of *ACT* used as an internal control [29]. The means and standard deviations were calculated from three technical replicates. Three biologically independent experiments were performed with similar results. The primer sets for qRT-PCR are listed in S1 Table.

## Supporting Information

S1 Table List of oligonucleotide primers used in this study. (XLS)
